# Photo‐Induced Charge State Dynamics of the Neutral and Negatively Charged Silicon Vacancy Centers in Room‐Temperature Diamond

**DOI:** 10.1002/advs.202308814

**Published:** 2024-03-12

**Authors:** G. Garcia‐Arellano, G. I. López‐Morales, N. B. Manson, J. Flick, A. A. Wood, C. A. Meriles

**Affiliations:** ^1^ Department of Physics CUNY‐City College of New York New York NY 10031 USA; ^2^ Department of Quantum Science and Technology Research School of Physics Australian National University Canberra ACT 2601 Australia; ^3^ CUNY‐Graduate Center New York NY 10016 USA; ^4^ Center for Computational Quantum Physics Flatiron Institute New York NY 10010 USA; ^5^ School of Physics The University of Melbourne Parkville VIC 3010 Australia

**Keywords:** charge state dynamics, diamond, Silicon vacancy centers

## Abstract

The silicon vacancy (SiV) center in diamond is drawing much attention due to its optical and spin properties, attractive for quantum information processing and sensing. Comparatively little is known, however, about the dynamics governing SiV charge state interconversion mainly due to challenges associated with generating, stabilizing, and characterizing all possible charge states, particularly at room temperature. Here, multi‐color confocal microscopy and density functional theory are used to examine photo‐induced SiV recombination — from neutral, to single‐, to double‐negatively charged — over a broad spectral window in chemical‐vapor‐deposition (CVD) diamond under ambient conditions. For the SiV^0^ to SiV^‐^ transition, a linear growth of the photo‐recombination rate with laser power at all observed wavelengths is found, a hallmark of single photon dynamics. Laser excitation of SiV^‒^, on the other hand, yields only fractional recombination into SiV^2‒^, a finding that is interpreted in terms of a photo‐activated electron tunneling process from proximal nitrogen atoms.

## Graphical abstract

Silicon vacancy (SiV) centers in diamond are candidates for quantum networks but exhibit charge instability under illumination. This work uses variable‐wavelength infra‐red excitation to characterize charge transfer from SiV^0^ to SiV^−^ and from SiV^−^ to SiV^2−^. These processes occur via different mechanisms, namely, single‐photon recombination and electron tunnelling from a nitrogen donor, findings supported by density functional theory modeling.

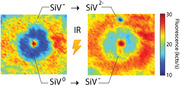

## 1 Introduction

Optically active spin qubits in semiconductor materials have emerged as attractive candidates for quantum information processing and sensing due to the high level of control achievable over single and coupled spins in a variety of solid‐state hosts.^[^ ^1^, ^2^, ^3^ ^]^ Among the most promising and widely studied systems are color centers in diamond, the nitrogen‐vacancy (NV), and silicon‐vacancy (SiV) centers arguably being the best‐known examples. The negatively charged NV (here denoted as NV**^‒^**) offers long spin coherence times at room temperature and is thus optimally suited for spin‐based sensing^[^ ^4^, ^5^ ^]^ but its broad fluorescence spectrum renders it impractical in quantum protocols requiring the generation of identical photons. By contrast, the negatively charged SiV (or SiV**^‒^**) possesses narrow optical emission and high quantum efficiencies^[^ ^6^, ^7^, ^8^, ^9^ ^]^— desirable for integration into photonic systems^[^ ^10^, ^11^, ^12^, ^13^ ^]^ — but its spin properties are poor. Bridging the gap between the two is the neutral silicon vacancy center (SiV^0^), simultaneously combining narrow optical emission (with zero phonon line at 946 nm) and spin coherence times approaching one second at cryogenic temperatures.^[^ ^14^, ^15^, ^16^, ^17^ ^]^ The attractive optical and spin features of SiV^0^ are tempered by challenges associated with generating and stabilizing the neutral charge state, typically requiring careful materials engineering of the host diamond. Formation of SiV^0^ can be favored by shifting the Fermi level by chemical means, either by boron doping during crystal growth, or through hydrogen termination of the diamond surface;^[^ ^15^, ^16^, ^17^, ^18^ ^]^ an alternative possibility is to use above‐bandgap UV excitation.^[^ ^19^ ^]^ Exactly how the optical or spin properties of the defect are modified by these processes is generally not clear. Recently it was shown that SiV^0^ can be generated in pristine CVD diamond by two‐step capture of holes diffusing from remote NV centers subjected to charge cycling under green excitation.^[^ ^20^, ^21^ ^]^ This approach is convenient in that it allows one to examine the charge dynamics of SiV^0^ in the absence of the additional complexity created by proximity to dopants or the host crystal surface.

Here, we first investigate the population dynamics of SiV^0^ centers with wavelengths in the range 720–874 nm. While SiV^0^ is optically dark at room temperature, we infer its charge state dynamics by measuring the SiV**^‒^** fluorescence levels upon SiV^0^ recombination. For all studied wavelengths, we find a linear growth of the recombination rate with laser power, indicative of a single photon process. Informed by DFT, we interpret the SiV^0^ ↔ SiV^−^ recombination as the photo‐injection of an electron from the diamond valence band into the SiV^0^ excited state. For the same infra‐red (IR) excitation window, we also study the dynamics of the negatively charged silicon‐vacancy center as it transitions to the dark charge state corresponding to SiV^2−^. Unlike SiV^0^, we find this transformation manifests as a non‐exponential time evolution of the observed fluorescence, difficult to account for in a framework of an isolated color center. Instead, numerical and ab initio modeling hint at a more complex process where photoexcitation of SiV^−^ stimulates electron tunneling from a proximal substitutional nitrogen (N) impurity.

## 2 Results and Discussion

### 2.1 Experimental Protocol

The sample examined here is a [100] CVD‐grown diamond with a nitrogen concentration of 3 ppm^[^ ^22^ ^]^ and estimated SiV and NV concentrations ≈0.3 and 0.03 ppm, respectively. Along with nitrogen, silicon is a common contaminant in lower‐grade CVD diamond, typically incorporated during crystal growth due to etching of the reactor quartz windows. Throughout the experiments, a custom‐made multi‐color scanning confocal microscope with 532 and 632 nm laser paths was used for excitation and detection of SiV centers. A tunable Ti:Sa laser running in continuous wave (cw) mode serves as the third laser source for illumination at variable IR wavelengths in the range 700–1000 nm. A band pass filter was used to limit photon collection to a narrow window around the SiV^−^ zero phonon line (ZPL) at 737 nm (see Section S1, Supporting Information, for additional experimental details).

Characterizing the charge dynamics of SiV centers under optical excitation is challenging because, out of the three SiV charge states found in most CVD diamond samples — namely the neutral, single‐, and double‐negatively charged states — only SiV^−^ exhibits significant room‐temperature photoluminescence (in the form of a sharp peak at 737 nm and a weak phonon sideband^[^ ^23^ ^]^). Unlike the NV center, where absence of NV^−^ fluorescence indicates NV^0^ presence with near unitary fidelity,^[^ ^24^ ^]^ careful steps must be undertaken to unambiguously differentiate the optically inactive SiV^2−^ from SiV^0^. In the same vein, NV center emission leaking into the SiV^−^ ZPL band as well as rapid hole and electron injection from NVs during charge state initialization further complicate measurements.^[^ ^22^, ^25^ ^]^

**Figure 1:**
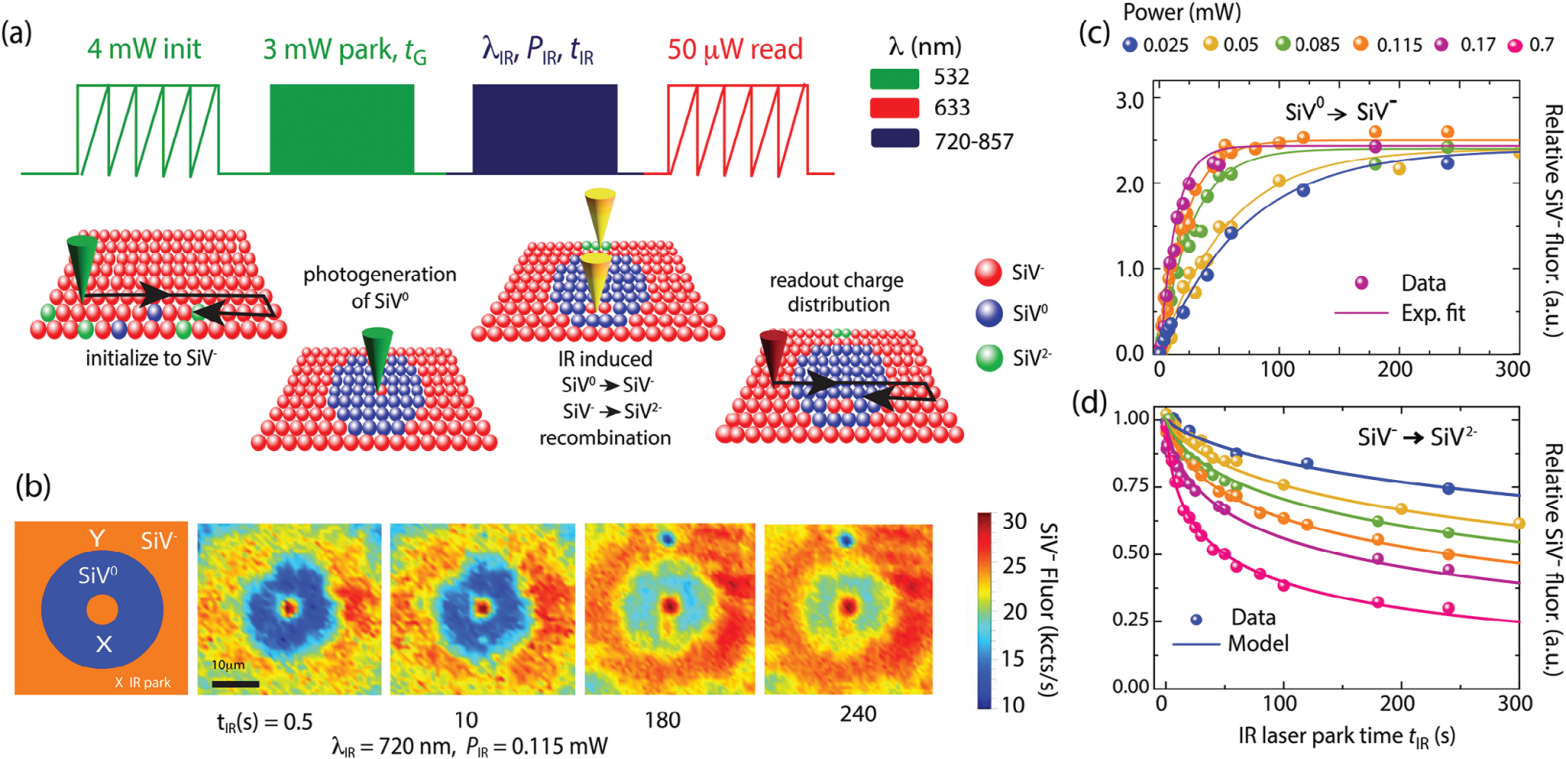
Initialization and detection of SiV^0^ and SiV^‒^ recombination under IR illumination. a) Experimental protocol. Following a 532 nm laser scan to produce an SiV^‒^‐rich background (5 mW, 1 ms dwell time per pixel, green zigzag in the diagram), we generate an SiV^0^‐rich halo via a 3 s, 532 nm park (solid green block). Setting a tunable Ti:Sa laser to a variable wavelength λ_IR_ and power *P*_IR_, we successively park the beam at two locations, X and Y, respectively in the SiV^0^‐ and SiV^‒^‐rich areas of the crystal for a variable time *t*_IR_ (solid blue block). Lastly, we image the resulting charge distribution via a weak red laser scan (632 nm at 50 µW, red zigzag). b) Example confocal images upon application of the protocol in (a) for variable time *t*_IR_. In this instance, λ_IR_ = 720 nm and *P*_IR_ = 0.115 mW; each image is an averaged composite of 4 scans per park time to account for imperfect initialization and power drifts of the Ti:Sa laser beam (<5%). c) Integrated SiV^−^ fluorescence (solid circles) at point X as a function of park time *t*_IR_ for variable laser powers under 720 nm excitation; solid lines are exponential fits. d) Integrated SiV^−^ fluorescence (solid circles) versus *t*_IR_ at point Y; solid lines represent fits to a model of nitrogen‐assisted electron tunneling (see below). In (c) and (b), a.u.: arbitrary units.

**Figure** **1**a schematically lays out the experimental protocol: A 532 nm beam was first scanned across an (80 µm)^2^ plane with the laser power (5 mW) and dwell time (1 ms) chosen so as to attain optimal SiV**^‒^** formation;^[^ ^22^ ^]^ the plane lies ≈5‒10 µm below the diamond surface, which eliminates the possibility of surface‐induced charge state effects. To produce an SiV^0^‐rich area within the plane, the green laser was subsequently parked at the center of the scanning range for a variable interval *t*_G_. Charge cycling of coexisting NV centers during this time results in the generation of free electrons and holes diffusing away from the illumination region. Preferential hole capture gradually transforms SiV^−^ — as well as any remaining SiV^2−^ — into SiV^0^. Note that the pattern (detected after a 50 µW, 632 nm scan) appears in the form of a “dark halo” since SiV^0^ photoluminescence (centered at 946 nm) is too weak for room temperature detection.

To characterize SiV^0^ recombination, the IR laser was parked at a point within the SiV^0^ pattern (marked with an “X” in the left schematic of Figure 1b) for a time *t*_IR_. For a given IR wavelength and power (respectively, denoted as λ_IR_ and *P*_IR_ in Figure 1b), we observe a gradual local conversion of SiV^0^ into SiV^−^ , here manifesting through the appearance of a bright spot in the otherwise dark SiV^0^ region (see Section S2, Supporting Information, for images at all other wavelengths). For the case at hand (λ_IR_ = 720 nm), Figure 1c illustrates the measured fluorescence change as a function of *t*_IR_ for variable IR laser power. To compensate for a change in the halo fluorescence (increasing at longer park time due to scattering of the IR beam), every data point in the plot was extracted from the difference between the SiV^−^ fluorescence at site X and the fluorescence from another reference site within the halo not directly exposed to IR. In all cases an exponential behavior could be seen whose dependence with laser power and wavelength provides key clues to interpret the underlying recombination mechanism, as shown in the next section.

Conveniently, the same experimental protocol could be used to probe photo‐induced SiV^−^ recombination into SiV^2−^ except that in this case the IR beam was parked at point “Y” in the SiV^−^‐rich section of the plane (see left‐hand side schematics in Figure 1b). Unexpectedly and in contrast to the previous case, infra‐red excitation leads here to a reduction of SiV^−^ fluorescence, revealed in Figure 1b by the appearance of a beam‐sized, dark spot in the otherwise bright SiV^−^ region. Figure 1d captures the recombination of SiV^−^ under 720 nm excitation  1d (where, as before, changes in the initial brightness were corrected by subtracting a suitable unaffected reference). The observed SiV^−^ bleaching — evolving non‐exponentially to reach a fractional level that depends on the applied laser power (and wavelength) — is somewhat surprising, as the IR photon energy (< 1.7 eV) is well below the charge transition threshold to SiV^2−^ (2.1 eV),^[^ ^26^, ^27^ ^]^ an intriguing response addressed below in Section 4, with the help of numerical and ab initio modeling.

### 2.2 Recombination Dynamics of SiV^0^

**Figure 2:**
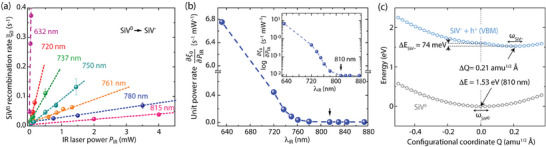
Recombination of SiV^0^. a) SiV^0^ recombination rate, ξ_0_, as a function of the IR laser power for variable wavelength. Dashed lines are linear fits. b) Unit power SiV^0^ recombination rates as derived from the linear fits in (a) as a function of wavelength. From the logarithmic plot (upper right insert), we determine the activation energy to be 1.53(5) eV (black arrow). c) Configurational coordinate diagram for SiV^‒^ + h^+^ recombination into SiV^0^. The value of 1.53 eV corresponds to the estimated ionization threshold, while 74 meV is the energy dissipated due to SiV^‒^ relaxation.

Analysis of the observed SiV^0^ fluorescence with applied IR excitation (Figure 1c above, see also Section S2, Supporting Information) allows us to identify the underlying recombination mechanism. **Figure** **2**a shows the measured recombination rate — extracted from a single exponential fit — as a function of laser power for each of the wavelengths studied in this work. We observe in all cases a linear dependence on light intensity, characteristic of a single photon process. Using the slopes in each data set to calculate the rate coefficients per unit laser power as a function of λ_IR_, we determine the threshold recombination energy as 1.53(5) eV (black arrow in Figure 2b). We can qualitatively interpret this process as the optical injection of an electron from the valence band into the excited state of SiV^0^ followed by relaxation into the SiV^−^ ground state.

To validate the dynamics at play, we model SiV^−^ **↔** SiV^0^ recombination using DFT. By obtaining the adiabatic potential energy surface (PES) for this process (Figure 2c), we find that the ionization threshold for SiV^0^ into SiV^−^ and a hole (h^+^) in the valence band maximum (VBM) matches very well the onset of the experimental curve in Figure 2b. The atomic relaxation for this process appears to be rather weak, characterized by small reconfiguration and energy changes (respectively, Δ*Q*  =  0.21 amu^1/2^Å, and Δ*E*  =  74 meV), which, in turn, suggest weak phonon interactions.

### 2.3 Recombination Dynamics of SiV^‒^

We now turn to studying the observed time evolution of the SiV^−^ fluorescence, decaying under IR illumination to reach intermediate values dependent on both laser power and wavelength. Adding to the markedly non‐exponential time response (see below), this incomplete recombination is difficult to rationalize in a framework that only takes SiV^−^ into account, hence suggesting a more complex scenario. One possibility is to consider the impact of coexisting impurities, substitutional nitrogen being the most natural choice. Known to exist both in neutral and positively charged forms (respectively denoted N^0^ and N^+^), electron transfer to and from substitutional nitrogen has already been invoked to understand charge state interconversion of NVs under continuous illumination in N‐rich samples.^[^ ^28^ ^]^ In the same vein, a charge transfer process involving pairs of proximal NVs and nitrogen — transforming as (NV**^‒^**)^*^ + N^+^ ↔ NV^0^ + N^0^ under green light — seems to underlie recent NV spectroscopy data at varying laser powers^[^ ^29^ ^]^ (the asterisk denotes the first excited state).

**Figure 3:**
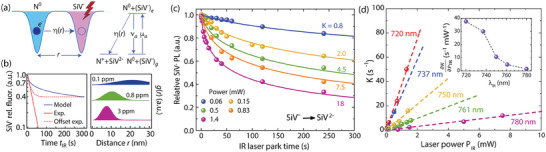
Nitrogen‐assisted recombination of SiV^‒^. a) Schematics of SiV^‒^ recombination. Under optical excitation, the donor electron from a proximal nitrogen tunnels into SiV^‒^. Since IR illumination does not excite N^0^, the SiV–N pair can be seen as a three‐level system undergoing a photo‐induced one‐directional transformation into N^+^ + SiV^2‒^; the notation follows that in the main text. b) (Left) Modeled SiV^‒^ relative fluorescence assuming *K*  =  40 s^−1^; for comparison, the plot includes a purely exponential decay with the same initial slope (solid red trace) and an exponential decay with an offset adapted to match the end value recorded over the measurement window (dashed red trace). (Right) Calculated nearest‐neighbor probability distribution for three example nitrogen concentrations; the blue trace on the left plot uses the distribution corresponding to 3 ppm and an effective tunneling radius *r*_0_ = 1 nm. c) Normalized SiV^‒^ fluorescence decay as a function of time after a 720‐nm laser parking in the SiV^‒^‐rich region for different laser powers. Solid lines are fits using Equation 3 in the main text. d) Effective SiV^‒^ recombination rate *K* as derived from (c) as a function of laser power for variable IR wavelength; dashed lines are linear fits whose slopes, $\frac{\partial K}{\partial P_{\mathrm{IR}}}$, we plot in the insert as a function of λ_IR_.

Here we posit that SiV^−^ recombination arises from tunneling of an electron between a photo‐excited SiV^−^ and a nearby N^0^, without requiring photoionization of the nitrogen (see **Figure** **3**a). We start by constructing a phenomenological model using rate equations to describe the observed charge dynamics. We consider a silicon‐vacancy and a substitutional nitrogen separated by a distance *r*, and assume that only SiV^−^ undergoes optical excitation under IR illumination (from the ground (*g*) to its first excited state (*e*), see Section S3, Supporting Information). This scenario is the most reasonable as the IR photon energies are below the 2.2 eV threshold where significant N^0^ photoionization onset occurs.^[^ ^30^, ^31^, ^32^ ^]^ We note this is significantly above the N^0^ donor level (1.7 eV) due to the additional energy required to redistribute the position of N and C atoms following loss of an electron.^[^ ^33^ ^]^ On the other hand, IR excitation below the SiV–ZPL is facilitated by anti‐Stokes absorption.^[^ ^34^ ^]^

Then, the rate equations that describe the SiV**^‒^** ($X_{\mathrm{SiV}}^{g,e})\;$and SiV^2^ **^‒^** ($X_{\mathrm{SiV}}^{2-})$ populations in the simultaneous presence of IR excitation and tunneling are given by

(1a) $$\frac{dX_{\mathrm{SiV}}^{g}}{dt}=-\mu_{\mathrm{SiV}}X_{\mathrm{SiV}}^{g}+\nu_{\mathrm{SiV}}X_{\mathrm{SiV}}^{e}$$

(1b) $$\frac{dX_{\mathrm{SiV}}^{e}}{dt}=\mu_{\mathrm{SiV}}X_{\mathrm{SiV}}^{g}-\nu_{\mathrm{SiV}}X_{\mathrm{SiV}}^{e}-\eta \left(r\right)X_{N}^{0}X_{\mathrm{SiV}}^{e}$$

In the above formulas, µ_SiV_, ν_SiV_ respectively represent the SiV^−^ IR excitation and relaxation rates, $X_{N}^{0}=1-X_{N}^{+}$ denotes the N^0^ population, and η(*r*) is the probability per unit time characterizing the electron transfer as a function of distance; we assume $\eta \left(r\right)=Ce^{-r/r_{0}}$ where *C* is a constant and *r*_0_ represents an effective tunneling radius.^[^ ^35^ ^]^ Since, in general, µ_SiV_,ν_SiV_ ≫ η, we can conveniently isolate the dynamics describing tunneling and recast Equations 1a and 1b under IR illumination as:

(2) $$\frac{d\left(1-X_{\mathrm{SiV}}^{2-}\right)}{dt}=-\eta \left(r\right)\frac{\mu_{\mathrm{SiV}}\xi_{N}}{\mu_{\mathrm{SiV}}+\nu_{\mathrm{SiV}}}{\left(1-X_{\mathrm{SiV}}^{2-}\right)}^{2}$$

where $1-X_{\mathrm{SiV}}^{2-}=X_{\mathrm{SiV}}^{g}+X_{\mathrm{SiV}}^{e}$ represents the SiV**^‒^** population at a given time (our observable, see schematics in Figure 1a). We use in Equation 2 the expression $X_{N}^{0}=\xi_{N}\left(1-X_{\mathrm{SiV}}^{2-}\right)$, where ξ_N_ = ξ_N_(λ_IR_,*P*_IR_) is, in general, a function of laser wavelength and power. This relation effectively ties the N charge state at a given time *t*_IR_ to that of the proximal SiV and is valid herein assuming the nitrogen concentration is sufficiently low (Section S3, Supporting Information). Solving Equation 2, we find:

(3) $$\bar{\left(1-X_{\mathrm{SiV}}^{2-}\right)}\left(t_{\mathrm{IR}}\right)=4\pi \int_{0}^{\infty }\frac{g\left(r\right)}{1+K\;t_{\mathrm{IR}}\;e^{-r/r_{0}\;}}r^{2}dr$$

where the upper bar denotes an average over all SiV–N pair distances, *g*(*r*) is a weight given by the nearest‐nitrogen probability distribution (itself a function of the nitrogen concentration, see Figure 3b), and *K*  =  *K*(λ_IR_,*P*_IR_) = $\frac{C\;\mu_{\mathrm{SiV}}\xi_{N}}{\mu_{\mathrm{SiV}}+\nu_{\mathrm{SiV}}}\;$is a fitting parameter dependent on the operating laser wavelength and power.

Adding to the results in Figure 1d, the solid circles and lines in Figure 3c respectively show the relative SiV^−^ fluorescence as a function of time for λ_IR_ = 750 nm and the calculated fits using Equation 3 assuming *r*_0_ = 1 nm, as seen for NV centers;^[^ ^36^ ^]^ in all cases, we set the nitrogen concentration at 3 ppm, consistent with prior observations in this same sample crystal.^[^ ^22^ ^]^ Comparison between the measured and calculated SiV response shows good agreement (see also Section S2, Supporting Information for the complete data set). Importantly, Equation 3 captures the non‐exponential response with the IR illumination time as well as the fractional SiV^−^ charge state conversion over the finite duration of the experiment, a combined consequence of the non‐linearity of Equation 2 and the varying contributions from pairs separated by growing distances (see also left plot in Figure 3b).

Figure 3d shows the extracted *K* values as a function of laser power, showing a linear dependence for all observed wavelengths. To interpret this observation, we note that in the low laser intensity limit — where ν_SiV_ ≫ µ_SiV_∝*P*_IR_, expected here — Equation 3 predicts *K*∝ξ_N_µ_SiV_, which implies a linear dependence with laser power provided ξ_N_ is insensitive to the excitation intensity.

The insert in Figure 3d shows the extracted slope, $\frac{\partial K}{\partial P_{IR}}$, as a function of λ_IR_; we find the process activates near the SiV^−^ ZPL, consistent with a charge transfer model where SiV^−^ excitation is a necessary condition.^[^ ^32^ ^]^ These observations alone, however, are insufficient to validate the energetics at play or understand the microscopic mechanisms driving electron tunneling from N^0^; we tackle these key aspects immediately below with the help of DFT.

### 2.4 Atomistic Modeling of Electron Transfer

**Figure 4:**
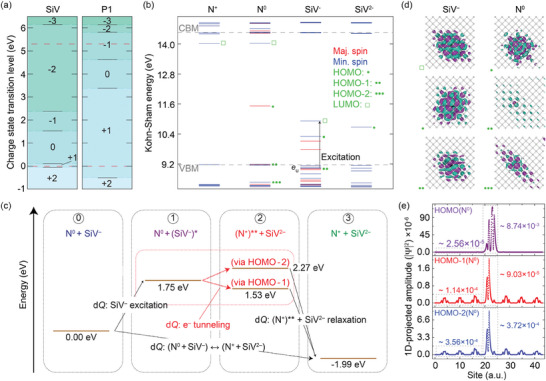
Ab initio modeling of N‐mediated SiV^‒^ recombination. a) Thermodynamic charge‐state transition levels for (left) SiV^‒^ and (right) N centers. b) Spin‐resolved electronic levels of N and SiV centers for the charge states relevant to our model. We use a square and an asterisk to respectively highlight the highest‐occupied and lowest‐unoccupied states (HOMO and LUMO, respectively); multiple asterisks indicate other defect (or defect‐mixed) states, ordered by the energy difference with respect to their HOMO. c) Energy‐level diagram as derived from PES minima for different SiV/N excitation, ionization, and recombination pathways. All levels are given with respect to the N^0^ + SiV^‒^ configuration, which is taken as the initial state for the whole charge transfer process. For reference, the diagram also includes the nature of the coordinate reconfigurations along each step. d) Real part of the wavefunction for the states highlighted in (b), see side labels. Here, cyan is positive and purple is negative; black, red, and gray spheres represent Si, N and C atoms, respectively. e) Line‐cuts along the 〈111〉 axis of the wavefunction magnitude for the three highest‐energy occupied states of N^0^. The integrated values along this direction are included for comparison. The supercell size is 512 atoms in (a) and (b) and 2474 atoms in (d) and (e); all calculations are carried out at the HSE06 level.

We carry out all DFT calculations in a 512‐atom supercell via the VASP software package^[^ ^37^ ^]^ and using either the PBE^[^ ^38^ ^]^ or HSE06^[^ ^39^ ^]^ exchange‐correlation functionals (Section S5, Supporting Information). We begin by determining the charge state transition energies for SiV and substitutional N (**Figure** **4**a): In agreement with prior work,^[^ ^27^, ^28^, ^40^, ^41^, ^42^ ^]^ the states most likely involved in an N‐mediated recombination of SiV^−^ (other than the starting SiV^−^ and N^0^) are SiV^2−^ and N^+^, which we now make the focus of our analysis. From the DFT energy levels for these defect charge states (Figure 4b), it is worth highlighting a few key features: *i*) N^+^ has no occupied intra‐bandgap defect states, with its lowest‐unoccupied molecular orbital (LUMO) lying close to the conduction band minimum (CBM); *ii*) besides the ^“^donor electron” state — here corresponding to the highest occupied molecular orbital (HOMO) — N^0^ also features two electronic states below the valence band maximum (VBM) whose wavefunctions show small but non‐negligible defect‐like contributions, which we marked as “HOMO‐1” and “HOMO‐2”, *iii*) the ***e*_u_** state of SiV^−^ also lies within the valence band, and appears to have an energy slightly lower than that of the mixed HOMO‐1 state in N^0^; *iv*) photo‐excitation of SiV^−^ propels an ***e*_u_** electron of the spin minority channel into the LUMO state (***e*_g_**); *v*) all relevant defect states in SiV^2^ **^‒^** are occupied.

Building on the above observations, the charge transfer mechanism we propose starts with the optical excitation of SiV^−^ into (SiV^−^)^*^, a process that leaves behind an unoccupied ***e*_u_** state (stage 1 in Figure 4c). The crucial step in the charge transfer is carrier tunneling, occurring through the relocation of a HOMO‐1 or HOMO‐2 electron into the (now vacant) SiV ***e*_u_** orbital (stage 2); relaxation of the nitrogen impurity — transiently left in an excited state we refer to as (N^+^)^**^ — drives N^+^ into the ground state irreversibly, in the process trapping the system into the N^+^ + SiV^2 −^ state (stage 3).

A key feature in this mechanism is the nature of the states involved: Unlike the N^0^ donor electron state, the HOMO‐1 and HOMO‐2 orbitals are comparatively delocalized due to hybridization with valence band states, arguably facilitating electron tunneling from N^0^ to (SiV^−^)^*^. We gauge the spatial spread in Figure 4d, where we boost the size of the supercell from 512 atoms — used in all other calculations — to 2474 atoms, and determine the wavefunctions of all relevant orbitals. We compare line cuts for the N^0^ wavefunction magnitudes along the 〈111〉 axis in Figure 4e, and find that the two sub‐VBM orbitals feature non‐negligible amplitudes across the supercell. Importantly, the electron densities we calculate for the HOMO‐1 and HOMO‐2 orbitals in areas farther removed from the defect site are orders of magnitude larger than those of the N^0^ donor level, hence facilitating the charge transfer. On a related note, we also highlight the close alignment between the energies corresponding to the N^+^ + (SiV^−^)* and (N^+^)** + SiV^2−^ transients (see stages 1 and 2 in Figure 4c), which makes this type of transfer close to resonant.

Aside from the energetics and spatial characteristics of the electronic wave functions, one last factor of interest is the level of atomic reconfiguration in the charge transfer process. By looking at the adiabatic PES of alternative N^0^ ionization pathways, we find a stark contrast between the extent of atomic reconfiguration when ionized via its HOMO state or via “HOMO‐1” or “HOMO‐2”, where the net configurational displacement (d*Q*) is ≈20‐fold smaller (between 0.05‒0.06 amu^1/2^ Å), which facilitates the charge transfer. These latter displacements are also akin to those obtained for processes involving the SiV^−^, which for the most part remain relatively small (<0.25 amu^1/2^ Å, see also Sections S4 through S6, Supporting Information).

## 3 Conclusions

In summary, we presented a comprehensive data set on the photo‐induced charge state dynamics of SiV throughout the important energy range where both SiV^0^ and SiV^−^ recombination activate. Our experiments capitalize on the ability to initialize the SiV charge on demand, either by direct optical excitation or through capture of carriers photo‐generated non‐locally. Building on ab initio modeling, we gained a microscopic understanding and, with it, a unified view of the charge state dynamics at play. Specifically, we can associate SiV^0^ recombination to a one‐photon process involving the injection of an electron from the valence band. In a similar manner, our experimental and theoretical work on SiV^−^ recombination supports the notion of a photo‐activated electron transfer from a proximal nitrogen donor. Rather than the N^0^ donor electron, carrier transfer takes place from intra‐valence‐band orbitals whose energies are nearly aligned with that of the SiV^−^ electron undergoing optical excitation. Combined, these results are consistent with photoconductivity measurements of bulk samples,^[^ ^43^, ^44^ ^]^ recent observations of bound excitonic states^[^ ^17^ ^]^ in SiV^0^, as well as preceding ab initio calculations on SiV.^[^ ^26^, ^27^ ^]^

Given the growing interest on the use of SiV centers for quantum information processing and nanoscale sensing applications, our findings have implications for the design and operation of devices that capitalize on the SiV physical properties. For example, the charge transfer processes unraveled here could serve as integral precursor steps to implementing optimized photoelectric detection of silicon vacancy centers, and in turn, understanding the generation of photocurrent in diamonds containing unwanted silicon impurities.^[^ ^45^ ^]^ In the same vein, manipulation of SiV^−^ as a stable photon source will benefit from additional efforts in reducing the concentration of proximal donors. Interestingly, a strong suppression of blinking and spectral diffusion in single SiV^−^ has been recently seen upon prolonged exposure to blue light,^[^ ^46^ ^]^ suggesting that local ionization of N^0^ into N^+^ could be responsible for the observed charge state stabilization.

While our modeling is consistent with nitrogen impurities serving as the electron source for SiV^−^ recombination, additional work —, e.g., involving the systematic characterization of diamond samples with variable nitrogen content — will be needed to conclusively assign the nature of the donor point defect. The limit of higher nitrogen concentration is particularly interesting as it is likely to alter the condition ξ_*b*_ ≈1 valid herein, and correspondingly change the observed fluorescence time response with laser power and wavelength.

Different charge initialization protocols — relying on green laser scans or capture of carriers diffusing from a non‐local green park — can arguably lead to nitrogen ensembles with distinct charge state compositions. Future theoretical and experimental work should therefore address the impact of these changes on the SiV recombination dynamics, specifically concerning the ability to augment or suppress charge tunneling from N to SiV. Similarly, it will be important to contemplate charge states in nitrogen beyond N^0^ and N^+^ considered here. Of particular interest is N^−^, proposed as a transient charge state to interpret absorption spectroscopy results^[^ ^47^ ^]^ but possibly present as a metastable, long‐lived charge configuration in crystals with sufficiently low nitrogen concentration.^[^ ^48^ ^]^

Although the present experiments are limited to ambient conditions, similar responses are also likely at lower temperatures as the invoked charge transfer mechanisms — namely, one‐photon optical injection and electron tunneling — are expected to be largely temperature insensitive. Something equivalent can be said about extensions from SiV ensembles to individual color centers, provided the nitrogen concentration remains moderately high. Lastly, the ability to initialize SiV into the neutral state combined with tunable laser excitation slightly below the ionization threshold open opportunities to further examine the formation of bound excitonic states in the absence of detrimental dopants^[^ ^17^ ^]^ (previously required to stabilize SiV^0^).

## Conflict of Interest

The authors declare no conflict of interest.

## Supporting information

Supporting Information

## Data Availability

The data that support the findings of this study are available from the corresponding author upon reasonable request.
