# Supplementary material for: Photo‐Induced Charge State Dynamics of the Neutral and Negatively Charged Silicon Vacancy Centers in Room‐Temperature Diamond
Source: Adv Sci (Weinh). 2024 Mar 12;11(22):2308814. doi: 10.1002/advs.202308814 (PMC11165459; doi:10.1002/advs.202308814)
Supplement: Supplementary file 1 — Supporting Information [file ADVS-11-2308814-s001.pdf]

ADVANCED SCIENCE
Open Access

Supporting Information

for *Adv. Sci.*, DOI 10.1002/advs.202308814

Photo-Induced Charge State Dynamics of the Neutral and Negatively Charged Silicon Vacancy Centers in Room-Temperature Diamond

*G. Garcia-Arellano, G. I. López-Morales, N. B. Manson, J. Flick, A. A. Wood and C. A. Meriles**

SUPPLEMENTARY MATERIAL

Photo-induced ionization dynamics of the neutral and negatively charged silicon vacancy defect in CVD diamond at room temperature

G. Garcia-Arellano[1], G. López-Morales[1], N. B. Manson[4], J. Flick[1,2,3], A. A. Wood[5], and C. A. Meriles[1,2]

[1]Department of Physics, CUNY-City College of New York, New York, New York 10031, USA.

[2]CUNY-Graduate Center, New York, NY 10016, USA.

[3]Center for Computational Quantum Physics, Flatiron Institute, New York, NY 10010, USA.

[4]Department of Quantum Science and Technology, Research School of Physics, Australian National University, Canberra, A.C.T. 2601, Australia

[5]School of Physics, The University of Melbourne, Parkville VIC 3010 Australia

1. **Experimental methods: Multicolor confocal microscopy at room temperature.**
2. **SiV^0 and SiV^- ionization dynamics under near-IR excitation.**
3. **Nitrogen-assisted electron tunneling.**
4. **SiV^0 and SiV^- ionization dynamics in a different CVD sample.**
5. **Computational methods.**
6. **Configurational coordinate diagrams (CCDs) and electron transfer via N^0's highest occupied molecular orbital.**

1. Experimental methods: Multicolor confocal microscopy at room temperature

The experimental setup consists of a multi-color confocal microscope with two branches for visible and the infrared excitation (Figure S1). For the visible branch, we use a 40-mW laser at 532 nm sourced from a DPSS laser diode module (Thorlabs DJ532-40) and a 633-nm red light from a 70-mW laser diode (Thorlabs HL63163DG). Light from both sources is combined via a dichroic mirror (Thorlabs DMLP638) and coupled into a single-mode fiber. The output from the fiber is collimated with an NA = 0.5 achromatic objective lens (Olympus UMPlanFl N). The scanning confocal microscope uses a two-axis galvo steering mirror system (Thorlabs GVS002), a 4*f*-relay lens (f_1 = 100 mm, f_2 = 200 mm), and a NA = 0.4 microscope objective (Mitutoyo MPlan Apo 2) mounted on a one-axis piezo scanning stage [1]

For the near-infrared excitation, we use a Ti:Sapphire laser (Coherent Mira 900) tunable between 700 and 900 nm and run in cw-mode. The output beam of the laser is intensity controlled with an acousto-optic modulator (AOM, Isomet 1250 C-829A) and thereafter sent into a single-mode optical fiber. The output from the fiber, after collimation, is reflected off a motorized flip mirror on the transmission side of the dichroic mirror, that allows to switch between the near-IR excitation and readout. We control the beam intensity using a variable neutral density filter at the output of the fiber.

The light from the diamond is collected using the reverse optical path and subsequently separated from the excitation light by the dichroic mirror. To select regions of the PL spectrum we first use 700 nm and 650 nm long-pass filters to prevent red light leakage to the detection, and a 735-nm, 10-nm-wide band pass filter to select out the SiV^- zero-phonon line (ZPL). The PL is then focused using a 12.5-mm aspheric lens into a single-mode fiber (MFD = 10 μm) and directed into a single-photon counting module (SPCM, Excelitas SPCM-AQRH-14). Photon counts are recorded using a National Instruments NI-PCIe 6321 data acquisition card, which additionally supplies the voltage for driving the galvo scanning system, the AOM and motorized flip mirror.

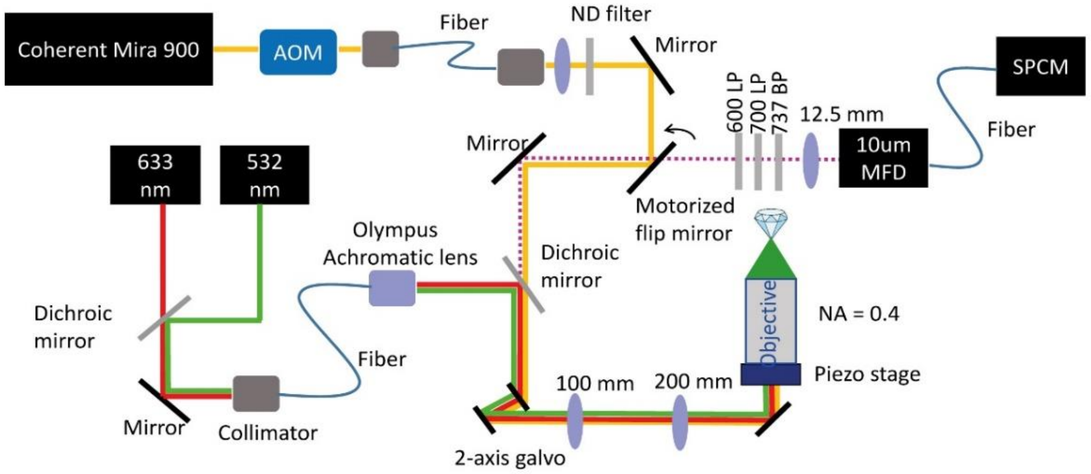

Figure S1. Multicolor confocal microscope used to study the recombination dynamics of SiV^- and SiV^0 under near-IR illumination.

2. SiV^0 and SiV^- ionization dynamics under near-IR illumination

In Figures S2-S6 (a) we present examples of confocal images obtained to study the ionization dynamics of the neutral (SiV^0) and negatively (SiV^-) charge states of the silicon vacancy defect at different wavelengths in the range 720-780 nm, under the protocol described in Fig. 1(a) of the main text. The IR laser is parked at the SiV^0 (X point) and SiV^- (Y point) as indicated in the

diagram of Fig. S2(a) for a variable park time t_{IR}. The wavelength, power and parking time are indicated in each of the figures.

Figures S2-S6 (b) present the integrated fluorescence (solid points) vs parking time obtained from the excitation of the photo-generated SiV^0 under near-IR illumination. The solid lines are exponential fits to the data. From each fit we extract the SiV^0 ionization rate (ξ_0) at a given power and we plot them in Fig. 2(a) of the main text. For all wavelengths, we observe a linear dependence with the power, a hallmark of a single photon process. To characterize the linear behavior, we have also plotted in Fig. (2b) of the main text the slope (or unit power rate $\frac{\partial \xi_0}{\partial P_{IR}}$) as a function of laser wavelength.

Similarly, Figures S2-S6 (c) show the integrated fluorescence data (solid points) vs parking time obtained from the excitation of SiV^- with near IR illumination. At all wavelengths in the range 720 - 780 nm we observe a non-exponential decay of the fluorescence. The solid lines are fits based on the nitrogen-assisted model described below (see section 3). The fitting parameter K extracted at each power is reported in Fig. 3(d) of the main text.

In order to determine the SiV^0 recombination threshold with a better accuracy, we performed additional measurements at wavelengths longer than 780 nm. Fig. S7 shows the integrated fluorescence (solid points) vs parking time, obtained when parking on the SiV^0 region (X point) with a) 633 b) 815 and c) 830, 844 and 874 nm illumination. The SiV^0 unit power rates for each wavelength are presented in Fig. 2(b) of the main text.

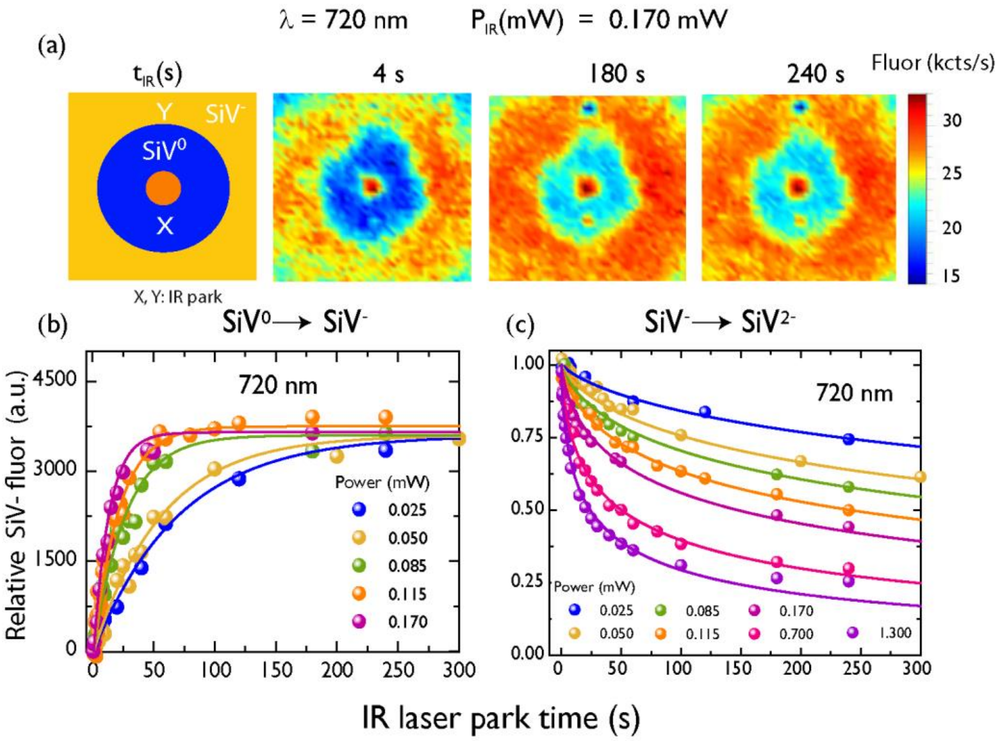

Figure S2: See captions below.

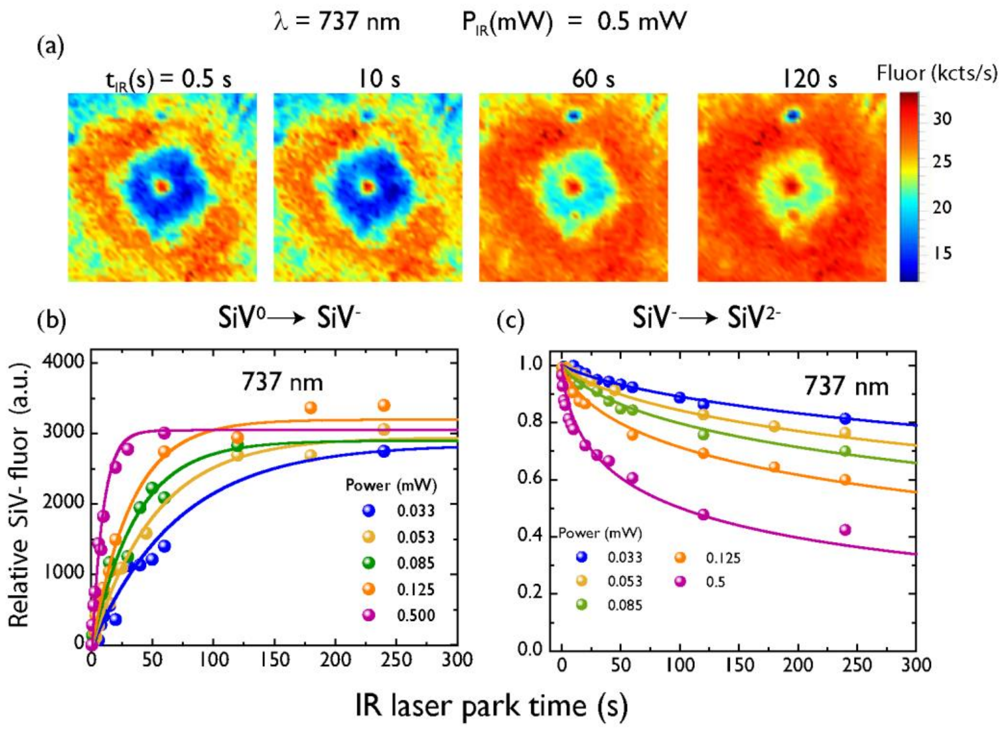

Figure S3: See captions below.

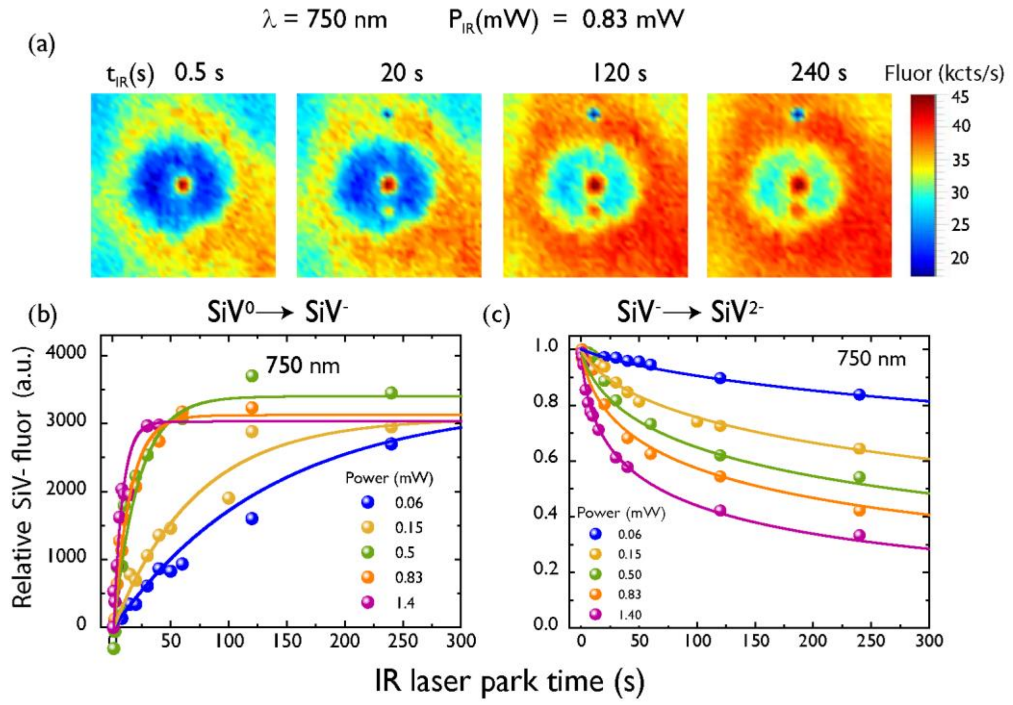

Figure S4: See captions below.

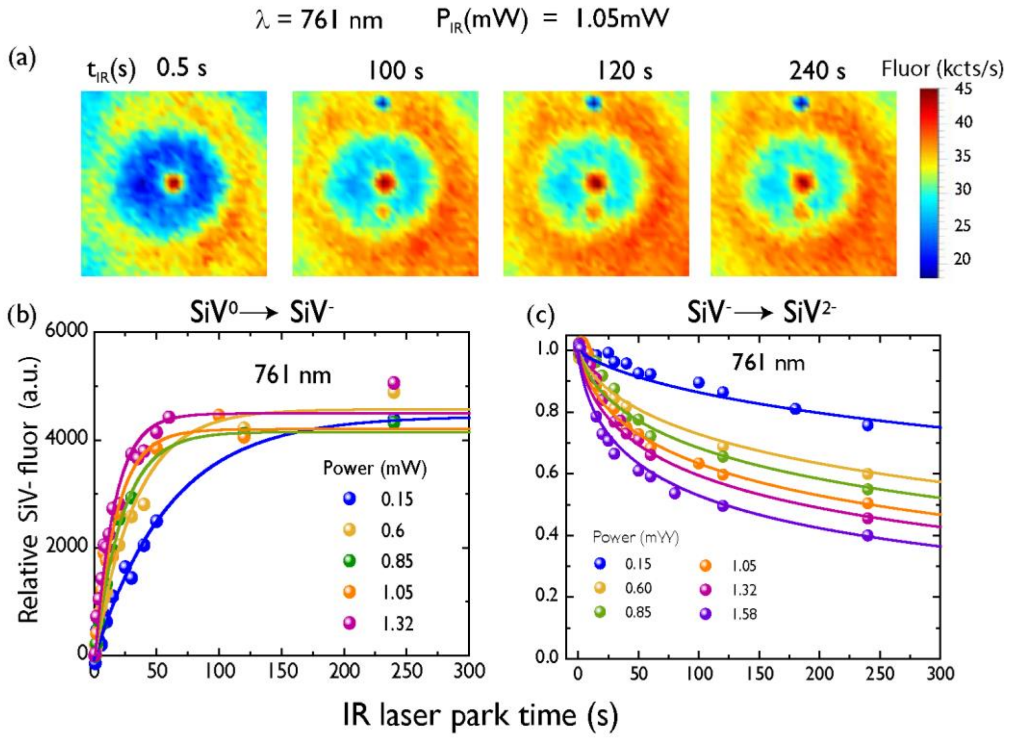

Figure S5: See captions below.

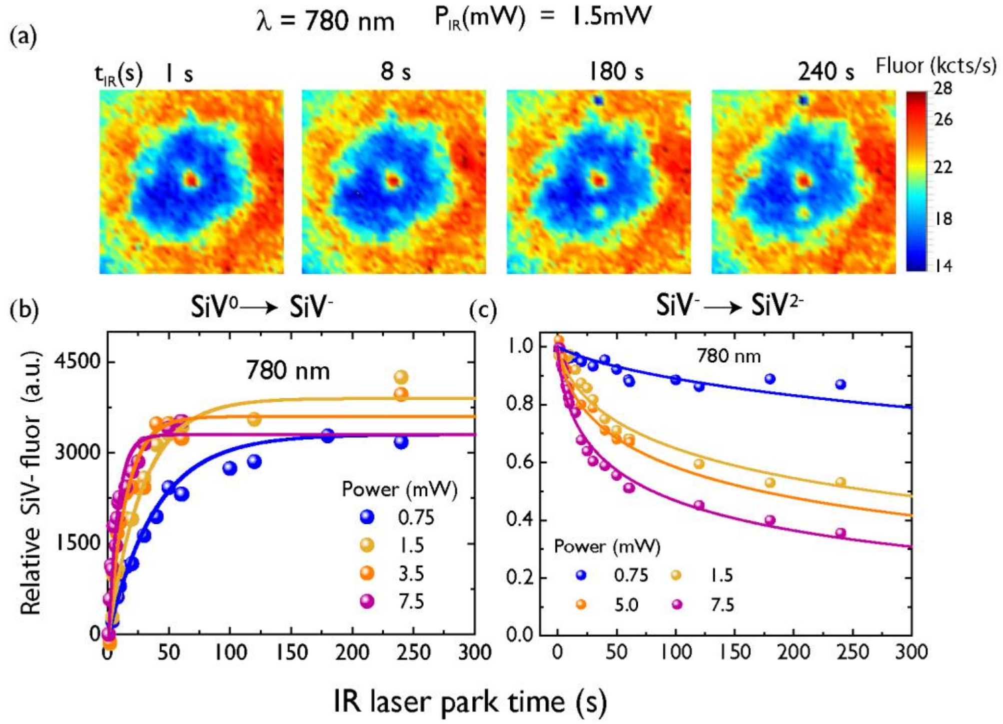

Figure S6: See captions below.

Figures S2- S6: (a) Confocal images obtained following the protocol described in Fig 1(a) of the main text. After photogeneration of the SiV^0 pattern, the IR laser is parked at the position X (SiV^0 region) and Y (SiV^- region) at different powers and wavelengths as indicated in the diagram of figure S2(a). (b) Integrated SiV^- fluorescence (solid points) versus parking time obtained at different powers of the Ti:Sa laser at the wavelength in the SiV^0 region (X point). The solid lines are exponential fits to the data The rate extracted from the fits at each wavelength is reported in Fig. 2(a) of the main text). (c) Integrated SiV^-fluorescence (solid points) versus parking time obtained at the SiV^- region. The solid lines are fits to the data using the nitrogen assisted electron tunneling model. (see section 3 below). The coefficient K extracted at each fit is reported in Fig. 3(d) of the main text.

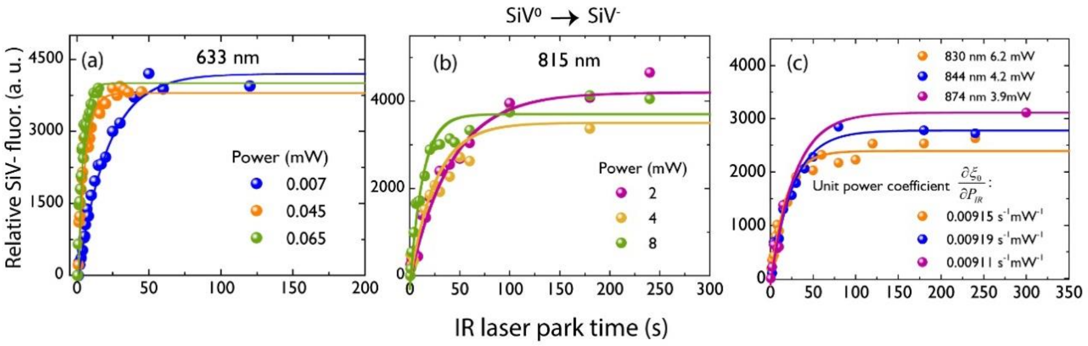

Figure S7: Integrated fluorescence (solid points) vs parking time obtained from the excitation of SiV^0 (X point) with a) 633nm, b) 815 nm and c) 830, 844, 874 nm illumination. The SiV^0 unit power rates are reported in Fig. 2(b) of the main text.

3. Nitrogen-assisted electron tunneling

We focus our attention now on modelling the recombination process of SiV^- into SiV^{2-} under near-IR illumination. We start by considering a defect pair comprising a negatively charged SiV and a neutral N separated by a distance r, and posit that SiV^- recombination arises from tunneling of the donor electron from N^0 during photoexcitation of SiV^-. Despite its nominally low ionization energy (~1.7 eV or, equivalently, 730 nm), we assume N^0 does not undergo excitation since its absorption cross section is low in the wavelength range relevant to these experiments (see Fig. 3(a) of the main text). The rate equations governing the dynamics can then be cast as

$$\frac{dX_{\mathrm{SiV}}^{g}}{dt} = -\mu_{\mathrm{SiV}} X_{\mathrm{SiV}}^{g} + \nu_{\mathrm{SiV}} X_{\mathrm{SiV}}^{e} \qquad \text{(S1a)}$$

$$\frac{dX_{\mathrm{SiV}}^{e}}{dt} = \mu_{\mathrm{SiV}} X_{\mathrm{SiV}}^{g} - \nu_{\mathrm{SiV}} X_{\mathrm{SiV}}^{e} - \eta(r) X_{\mathrm{N}}^{0}(\vec{r},t) X_{\mathrm{SiV}}^{e}(0,t) \qquad \text{(S1b)}$$

where X_{SiV}^{g} (X_{SiV}^{e}) represents the fractional SiV^- population in the ground (excited) state, μ_{SiV} and ν_{SiV} respectively denote the SiV^- optical excitation and relaxation rates, and $\eta(r)$ represents the unit time probability characterizing the electron transfer. Note that the probability of finding the proximal nitrogen in the neutral charge state, $X_{\mathrm{N}}^{0}(\vec{r},t)$, is, in general, a function of time t and its position $\vec{r}$ relative to the SiV (assumed at the origin). On the other hand, the SiV charge states satisfy the conservation equation $X_{\mathrm{SiV}}^{g} + X_{\mathrm{SiV}}^{e} + X_{\mathrm{SiV}}^{2-} = 1$ with X_{SiV}^{2-} denoting the fractional population in the SiV^{2-} charge state. Then, we recast Eq. S1(a) as

$$\frac{dX_{\mathrm{SiV}}^{g}}{dt} = -\left(\mu_{\mathrm{SiV}} + \nu_{\mathrm{SiV}}\right) X_{\mathrm{SiV}}^{g} + \nu_{\mathrm{SiV}}\left(1 - X_{\mathrm{SiV}}^{2-}\right) \qquad \text{(S2)}$$

Assuming that X_{SiV}^{2-} changes very slowly in comparison with the excitation and relaxation rates μ_a, ν_a, then the solution to Eq. S2 takes the form $X_{\mathrm{SiV}}^{g} = A + Be^{-\kappa t}$ with A, B, and κ given by $A = \frac{\nu_{\mathrm{SiV}}}{\mu_{\mathrm{SiV}} + \nu_{\mathrm{SiV}}}\left(1 - X_{\mathrm{SiV}}^{2-}\right)$, $B = \frac{\mu_{\mathrm{SiV}}}{\mu_{\mathrm{SiV}} + \nu_{\mathrm{SiV}}}\left(1 - X_{\mathrm{SiV}}^{2-}\right)$, and $\kappa = \mu_{\mathrm{SiV}} + \nu_{\mathrm{SiV}}$

We can now rewrite X_{SiV}^{g} as

$$X_{\mathrm{SiV}}^{g} = \left(1 - X_{\mathrm{SiV}}^{2-}\right) \frac{1}{\mu_{\mathrm{SiV}} + \nu_{\mathrm{SiV}}} \left(\nu_{\mathrm{SiV}} + \mu_{\mathrm{SiV}} e^{-(\mu_{\mathrm{SiV}} + \nu_{\mathrm{SiV}})t}\right). \qquad \text{(S3)}$$

Similarly, we express X_{SiV}^{e} as

$$X_{\mathrm{SiV}}^{e} = \left(1 - X_{\mathrm{SiV}}^{2-}\right) - X_{\mathrm{SiV}}^{g}$$

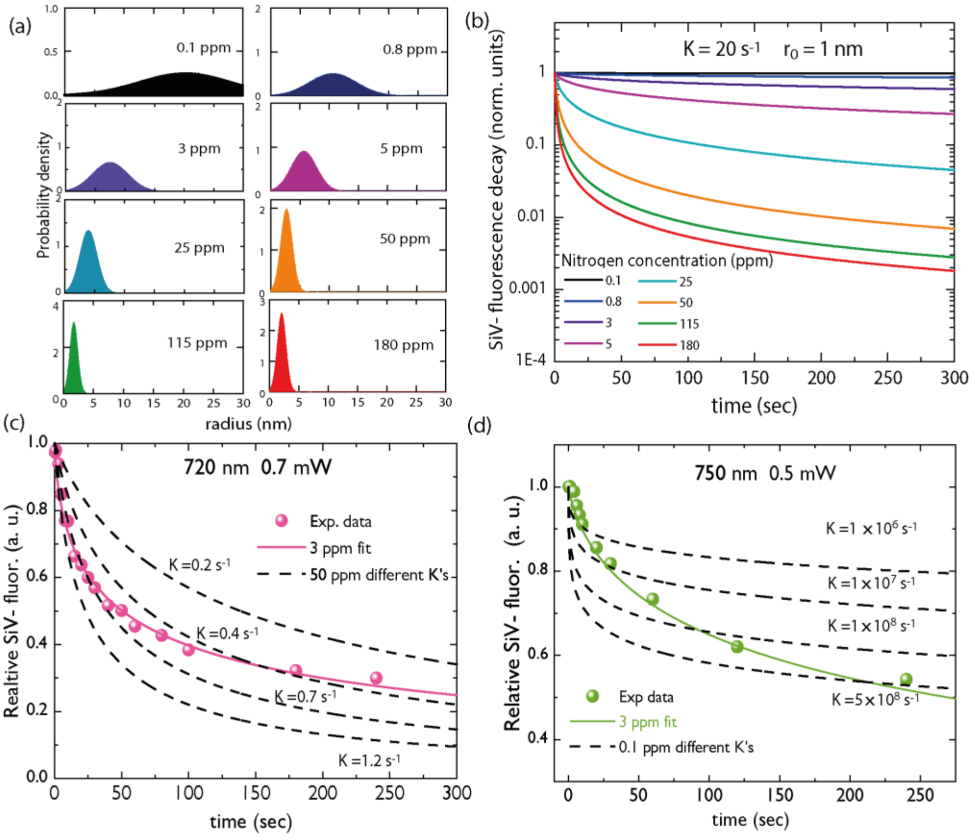

Figure S8: (a) Nearest-neighbor probability distribution for different nitrogen concentrations. (b) Calculated SiV^- fluorescence obtained from Eq. S8 assuming that the initial SiV^- concentration is fixed and illuminated under near-IR at fixed power for different nitrogen concentrations. (d) Comparison of the data at 720 nm and 0.7 mW (solid points) fitted using a nearest-nitrogen distribution corresponding to 3 ppm (solid pink line) and the calculated SiV^- dynamics with a nitrogen distribution of 50 ppm for different K values. (c) Comparison between the data at 750 nm and 0.5 mW (solid points) fitted using a nearest-nitrogen probability distribution corresponding to 3 ppm (solid green line) and the expected SiV^- dynamics with a nitrogen distribution of 0.1 ppm for varying K. The tunneling radius is fixed at $r_0 = 1$ nm.

$$= \left(1 - X_{\mathrm{SiV}}^{2-}\right)\frac{\mu_{\mathrm{SiV}}}{\mu_{\mathrm{SiV}}+\nu_{\mathrm{SiV}}}\left(1 - e^{-(\mu_{\mathrm{SiV}}+\nu_{\mathrm{SiV}})t}\right) \qquad \text{(S4)}$$

Adding Eqs. S1(a) and S1(b), we then obtain

$$\frac{d(1-X_{\mathrm{SiV}}^{2-})}{dt} = -\eta(r) X_{\mathrm{SiV}}^{e}(\vec{r},t) X_{\mathrm{N}}^{0}(\vec{r},t) = -\eta(r)\frac{\mu_{\mathrm{SiV}}}{\mu_{\mathrm{SiV}}+\nu_{\mathrm{SiV}}}\left(1 - X_{\mathrm{SiV}}^{2-}\right) X_{\mathrm{N}}^{0}(\vec{r},t), \qquad \text{(S5)}$$

where we assumed X_{SiV}^{e} (and X_{SiV}^{g}) take their time-independent limit values throughout the evolution, an approximation justified given that $(\mu_{\mathrm{SiV}}+\nu_{\mathrm{SiV}})^{-1}$ is a very short time scale compared to that defined by the inverse tunneling rate, η^{-1}. For excitation wavelengths above 720 nm, no ionization of N^0 has been reported, hence allowing us to write $X_{\mathrm{N}}^{0}(\vec{r},t) = \xi_{\mathrm{N}}\left(1 - X_{\mathrm{SiV}}^{2-}\right)$, where $\xi_{\mathrm{N}} = \xi_{\mathrm{N}}(\lambda_{\mathrm{IR}}, P_{\mathrm{IR}})$ is, in general, a function of laser wavelength and power. Here we assume that $\eta(r)$ decreases exponentially with the distance between the SiV^- and N^0, and is given by the exponential function $\eta(r) = Ce^{-r/r_0}$ with r_0 denoting the effective tunneling radius. The solution to Eq. (S5) is then

$$\left(1 - X_{\mathrm{SiV}}^{2-}(t)\right) = \frac{1}{1+K\, t_{\mathrm{IR}}\, e^{-r/r_0}}, \qquad \text{(S6)}$$

where $K(\lambda_{\rm IR}, P_{\rm IR}) = \frac{C\,\mu_{\rm SiV}\xi_{\rm N}}{\mu_{\rm SiV}+\nu_{\rm SiV}}$ is a fitting parameter dependent on the operating laser wavelength and power. Finally, if we consider an ensemble of pairs separated by a variable distance r, the fluorescence decay — governed by $(1 - X_{\rm SiV}^{2-}(t))$ — is given by the expression

$$\overline{(1 - X_{\rm SiV}^{-})} = 4\pi \int_0^{\infty} r^2 \frac{g(r)}{1+K\,t_{\rm IR}\,e^{-r/r_0}} dr, \tag{S7}$$

where the upper bar denotes an average over all SiV–N pair distances, and $g(r)$ represents the nearest neighbor probability distribution, in turn a function of the nitrogen concentration (Fig. S8(a)) [3]. In Fig. S8(b) we plot the expected SiV^- fluorescence decay at fixed power ($K = 20$ s^{-1}) and radius $r_0 = 1$ nm obtained from Eq. S7. As we can see, if the nitrogen concentration increases, a larger fraction of the SiV^- initial population is transformed into SiV^{2-} via electron tunneling.

To analyze the data shown in Figs. S2(c) to S6(c) we set the nitrogen concentration to 3 ppm, consistent with prior observations in this sample [2] and $\xi_b \sim 1$. As shown in Fig. 3(d) of the main text, the fitting parameter K exhibits a linear dependence with the power for all wavelengths in the range 720-780 nm. Consistent with this observation, we expect $\nu_{\rm SiV} \gg \mu_{\rm SiV} \propto P_{\rm IR}$ in the limit of low laser intensities, implying that $K \propto \xi_{\rm N}\mu_{\rm SiV}$ and thus linear with laser power (provided $\xi_{\rm N}$ insensitive to the excitation intensity).

In Figs. S8(c) and S8(d) we show the expected SiV^- fluorescence decay calculated using nitrogen concentrations one order of magnitude smaller (S8(c): 0.1 ppm) and one order of magnitude larger (S8(d): 50 ppm) than the one estimated in the sample (solid lines). As we can see nitrogen

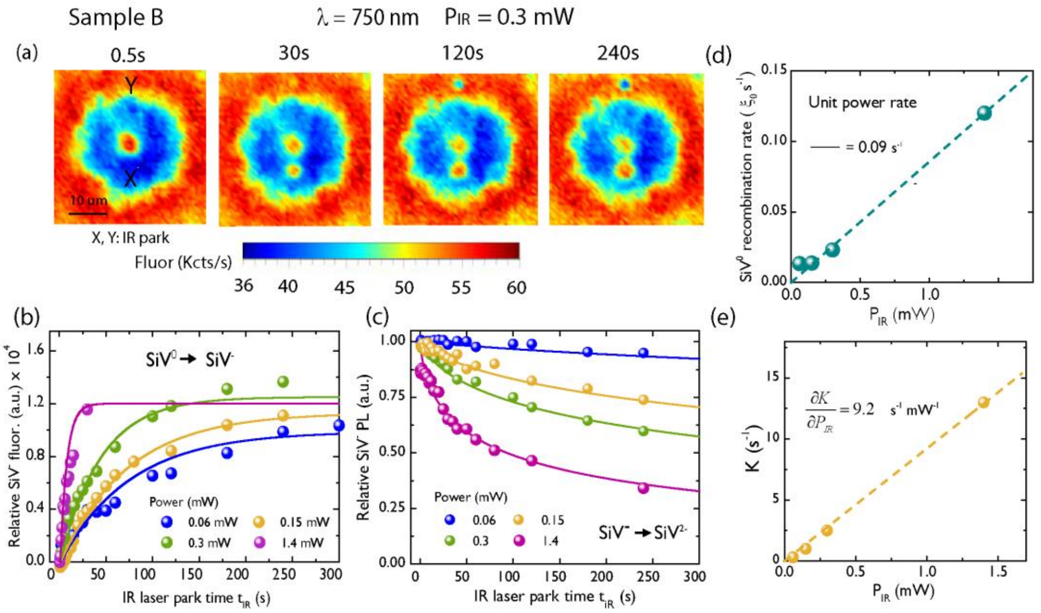

Figure S9. (a) Example of the confocal images upon application of the protocol in (a) for variable time in sample B. Each image is an averaged composite of 4 scans per park time to account for imperfect initialization and power drifts of the Ti:Sa laser beam (5%). (c) Integrated SiV^- fluorescence (solid circles) at point X as a function of park time for variable laser powers under 750 nm excitation; solid lines are exponential fits. (c) Integrated SiV^- fluorescence (solid circles) versus time at point Y; solid lines represent fits to the model of nitrogen-assisted electron tunneling (see section 3 of the supplementary material). In (b) and (c), a.u.: arbitrary units. (d) SiV^0 recombination rates and unit power rate extracted from the exponential fits show in (b). (c) coefficient K versus power obtained from the fit of the SiV^- to SiV^{2-} recombination upon the model of nitrogen-assisted electron tunneling.

concentrations different to the 3-5 ppm expected for this diamond crystal do not properly reproduce the experimental data.

4. SiV^0 and SiV^- dynamics in a different CVD sample

In this section we present ionization measurements for a different CVD sample (named sample B). The SiV and NV concentrations are 0.9 ppm and 10 ppb respectively [3]. Figure S9(a) shows the confocal images obtained under the protocol presented in the main text (Fig. 1(a)). The IR laser (750 nm) is parked in the SiV^0 region (X point) and SiV^- region (Y point) at different powers. Figure S9(b) shows the integrated fluorescence obtained after illumination of the SiV^0 region at different powers. The SiV^0 ionization rate at each power is presented in Fig. S6(d). The extracted unit power rate ξ_0 matches with the one found in the sample presented in the main text (see blue curve at 750 nm in Fig. 2(a) of the main text).

Figure S9(c) presents the integrated SiV^- fluorescence (solid circles) vs time at point Y. In agreement with the sample studied in the main text, we also observe a non-exponential decay of the SiV^- fluorescence under near-IR illumination. The solid lines represent fits to the model of nitrogen-assisted electron tunneling (see section 3) using a nitrogen concentration of 3 ppm. The coefficient K versus power obtained from the fit is presented in Fig. S9(e). The slope that characterizes the linear dependence of K with power is comparable to the one obtained in the sample studied in the main text.

5. Computational methods

All density functional theory (DFT) calculations presented herein are performed within the PAW method[4,5], using Perdew-Burke-Ernzerhoff (PBE)[6] and hybrid (range-separated) Heyd-Scuseria-Ernzerhoff (HSE06)[7] functionals to account for electronic exchange-correlation interactions during atomic relaxations and self-consistent (SCF) calculations, respectively. The potential-energy surfaces (PES) of the various processes considered herein are all derived within the adiabatic Born-Oppenheimer approximation[8-10]. For all excited-state PES, the atoms are displaced along the configurational coordinate while keeping constrained occupation of the electronic states via the so-called constrained DFT (cDFT) method[6,7]. For the plane-wave basis, we use a low kinetic-energy cut-off of 370 eV as employed in previous work[11], which has shown to yield relatively well-converged results. All equilibrium defect structures are obtained by embedding the necessary impurities/vacancies in a 4×4×4 (512-atom) diamond supercell (created from a volume-optimized diamond unit-cell) and relaxing the ions at constant volume until forces are below 10^{-3} eV/Å. The electronic loops are converged down to energy differences below 10^{-8} eV (precision of wave functions) for equilibrium configurations, and 10^{-4} for non-equilibrium (PES) configurations. All supercell calculations are employed through sampling of the Brillouin zone at the Γ-point only. In obtaining the equilibrium charge-state transition energies, and all related calculations involving charged defects, we employ the charge correction scheme proposed in Ref. [12] to alleviate the obtained total energies from supercell finite-size effects. To calculate wave function overlaps between different defect centers, we make use of independent, large (2474-atom) diamond supercells for each center, with the electronic loops converged to 10^{-9} eV energy-differences so as to ensure well-converged 'tails' (low-amplitude regions) of the defect wave functions[8].

6. Configurational coordinate diagrams and ionization/capture via the HOMO of N^0

This section considers alternative electron transfer pathways involving the HOMO states of N^0 and SiV^-. In Fig. S10, we show the full set of configurational coordinate diagrams (CCDs) with the transfer modelled by placing the electron in the diamond conduction band and adding the corresponding energy to the total energy of the system. In Fig. S10a, the calculated ionization

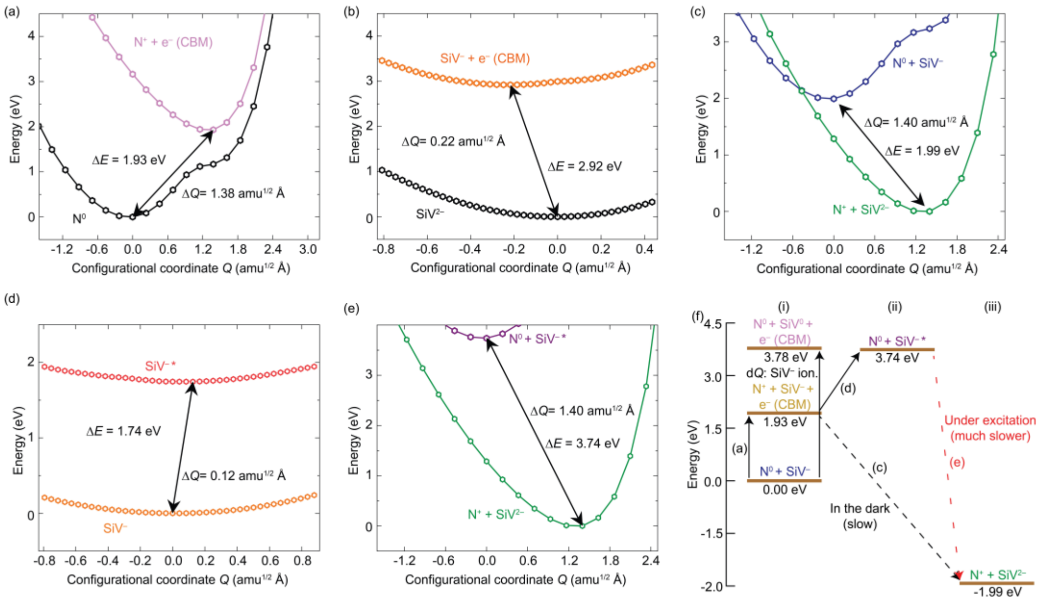

Figure S10. Charge transfer involving the N^0 donor electron. (a) Ionization of N^0 from the HOMO (via CBM). (b) Ionization of SiV^{2-} via the CBM. (c) CCD for the combined individual processes depicted in (a) and (b). Here, the energetic contribution from the e^- in the CB vanishes, putting the $N^+ + SiV^{2-}$ back at a lower energy compared to $N^0 + SiV^-$. (d) Optical excitation of SiV^-. (e) CCD for the combined individual processes depicted in (a) and (d). The energy difference between (c) and (e) correspond to the ZPL of SiV^-. (f) Energy-level diagram derived from the PES minima in (a–e), with the additional process of SiV^- ionization into the CB, included as a reference (the corresponding CCD is not shown for brevity). All levels are given with respect to the $N^0 + SiV^-$ configuration, which is taken as the initial state for the charge transfer process.

threshold of N^0 lies well within the range of experimental values reported for such a process (1.7-2.2 eV)[13,14]. We can also see that such ionization from HOMO causes a substantial reconfiguration of the N-defect structure. In contrast, the reconfiguration for the ionization of SiV^{2-} (or conversely, the electron capture by SiV^- via the conduction band) remains much smaller (Fig. S10b). Thus, in a recombination process involving the N^0 and SiV^- HOMOs (Fig. S10c), the rate will likely be dominated by the reconfiguration of N^0. For this process, we see that the PES curves cross, suggesting that electron transfer may happen in the dark (i.e., no SiV^- excitation required). However, because the reconfiguration is rather large, this process is likely phonon-mediated and slow. Additional experimental work will be required to see whether formation of SiV^{2-} via electron tunneling without light excitation can be seen experimentally; we note, however, that the localized nature of the donor electron orbital as compared to those of HOMO-1 and HOMO-2 would make this process more inefficient (see Fig. 4 and related paragraph in the main text).

In Fig. S10d, we show the CCD for the optical excitation of SiV^-, which (given its small experimental Huang-Rhys factor[15]), also has a small nuclear reconfiguration. Considering the SiV^- in the excited state (i.e., $(SiV^-)^*$) will shift the $N^0 + SiV^-$ curve in Fig. S10c by the Si^- ZPL energy (Fig. S10e), making the charge-transfer process even less likely. The resulting energy-level diagram summarizing the results in Fig. S10a-e is shown in Fig S10f. Here, the additional process of SiV^-ionization is also included (as a reference for comparison). With these results, we conclude that while charge-transfer from N^0 to SiV^- via the N^0 HOMO is plausible, it cannot explain the experimental observations discussed in the main text.

REFERENCES

[1] A. Wood, A. Lozovoi, Z.-H. Zhang, S. Sharma, G.I. López-Morales, H. Jayakumar, N.P. de Leon, C.A. Meriles, "Room temperature photo-chromism of silicon vacancy centers in CVD diamond", *Nano Lett.* **23**, 1017 (2023).

[2] S. Dhomkar, H. Jayakumar, P.R. Zangara, C.A. Meriles, "Charge dynamics in near-surface, variable-density ensembles of nitrogen-vacancy centers in diamond", *Nano Lett.* **18**, 4046 (2018).

[3] A. A. Wood, E. Lilette, Y. Y. Fein, V. S. Perunicic, L. C. L. Hollenberg, R. E. Scholten & A. M. Martin, *Nat. Phys.* **13**, 1070 (2017).

[4] G. Kresse, J. Furthmüller, "Efficient iterative schemes for ab initio total-energy calculations using a plane-wave basis set", *Phys. Rev. B* **54**, 11169 (1996).

[5] G. Kresse, D. Joubert, "From ultrasoft pseudopotentials to the projector augmented-wave method", *Phys. Rev. B* **59**, 1758 (1999).

[6] J. P. Perdew, K. Burke, and M. Ernzerhof, "Generalized gradient approximation made simple", *Phys. Rev. Lett.* **77**, 3865 (1996).

[7] J. Heyd, G.E. Scuseria, M. Ernzerhof, "Hybrid functionals based on a screened Coulomb potential", *J. Chem. Phys.* **118**, 8207 (2003).

[8] Ádám Gali, "*Ab initio* theory of the nitrogen-vacancy center in diamond", *Nanophotonics* **8**, 1907 (2019).

[9] G. Thiering, A. Gali, "*Ab initio* magneto-optical spectrum of group-IV vacancy color centers in diamond", *Phys. Rev. X* **8**, 021063 (2018).

[10] M. Bockstedte, F. Schütz, T. Garratt, V. Ivády, A. Gali, "Ab initio description of highly correlated states in defects for realizing quantum bits", *npj Quantum Materials* **31** (2018).

[11] J. Chou, Z. Bodrog, A. Gali, "First-principles study of charge diffusion between proximate solid-state qubits and its implications on sensor applications", *Phys. Rev. Lett.* **120**, 136401 (2018).

[12] C. Freysoldt, J. Neugebauer, C. G. Van de Walle, "Fully *ab initio* finite-size corrections for charged-defect supercell calculations", *Phys. Rev. Lett.* **102**, 016402 (2009).

[13] A. M. Ferrari, S. Salustro, F. S. Gentile, W. C. Mackrodt, R. Dovesi, "Substitutional nitrogen in diamond: A quantum mechanical investigation of the electronic and spectroscopic properties", *Carbon* **134**, 354 (2018).

[14]K. Iakoubovskii, G. J. Adriaenssens, "Optical transitions at the substitutional nitrogen centre in diamond" *J. Phys.: Condens. Matter* **12** L77 (2000).

[15] E. Neu, M. Fischer, S. Gsell, M. Schreck, C. Becher, "Fluorescence and polarization spectroscopy of single silicon vacancy centers in heteroepitaxial nanodiamonds on iridium", *Phys. Rev B* **84**, 205211 (2011).
